# Efficacy and safety of ciprofol for sedation/anesthesia in patients undergoing hysteroscopy: a prospective, randomized, non-inferiority trial

**DOI:** 10.1080/07853890.2025.2517820

**Published:** 2025-06-18

**Authors:** Shi Cheng, Haixuan Wu, Zhongqi Liu, Dan Liu, Minghui Cao, Ganglan Fu

**Affiliations:** Department of Anesthesiology, Sun Yat-Sen Memorial Hospital, Sun Yat-Sen University, Guangzhou, China

**Keywords:** Ciprofol, Propofol, Hysteroscopy

## Abstract

**Introduction:**

Propofol is the preferred sedative for painless hysteroscopy and other procedures due to its fast onset and short duration. However, its limitations, including injection pain, respiratory depression, blood pressure decline, and bradycardia, cannot be disregarded. Ciprofol, a new short-acting gamma-aminobutyric acid receptor agonist, has shown effectiveness and safety in painless gastrointestinal endoscopy. This study aims to demonstrate that ciprofol is not inferior to propofol in terms of sedation efficacy for painless hysteroscopy.

**Patients and methods:**

A randomized study was conducted with 124 women to evaluate the anesthetic effect of ciprofol during outpatient painless hysteroscopy. The primary outcome assessed was the success rate of hysteroscopy, while secondary indicators included induction and recovery time, injection pain, tidal volume and respiratory rate. Safety indicators comprised hypotension, hypoxemia, and sinus bradycardia.

**Results:**

A total of 124 patients were enrolled in the study, with 62 in each group. The success rate of hysteroscopy was 100% in both groups. Patients in the ciprofol group had higher diastolic pressure, pulse oxygen saturation levels and minute breathing during surgery than patients in the propofol group, but their induction time and recovery time were longer. The proportion of patients in the propofol group who reported pain during intravenous anesthesia was 41.935%, which was significantly higher than that of patients in the ciprofol group (1.613%).

**Conclusion:**

During painless hysteroscopy, ciprofol demonstrates non-inferiority to propofol in terms of anesthetic efficacy. Despite slightly longer induction and recovery times, ciprofol results in lower instances of injection pain and less impact on respiration and circulation compared to propofol.

**Trial number:**

Clinical trial Registration Identifier: NCT06172140.

## KEY MESSAGES

- During painless hysteroscopy, ciprofol demonstrates non-inferiority to propofol in terms of anesthetic efficacy. Ciprofol results in lower instances of injection pain and less disruption to patients’ respiration and circulation compared to propofol.

## 1. Introduction

As the need for medical comfort grows and anesthetic agents continue to improve, there is a rising number of outpatient painless gastrointestinal endoscopies, hysteroscopies, bronchoscopies, and other endoscopic examinations. The advancement of painless technology has significantly enhanced patient satisfaction and comfort. Anesthesiologists typically employ a mixture of opioid medications and propofol to achieve painless endoscopic procedures. Propofol is the top choice sedative for painless gastrointestinal endoscopy, hysteroscopy, and other procedures due to its quick onset and brief duration of action. However, its drawbacks, such as respiratory depression, decreased blood pressure, and bradycardia, should not be overlooked [1–4]. Additionally, many patients report experiencing injection pain [5,6]. As a result, there is a growing need for alternative anesthetic agents that are equally effective but have fewer adverse effects.

Ciprofol, a new short-acting gamma-aminobutyric acid (GABA) receptor agonist, received approval from the China National Medical Products Administration (NMPA) on 15 December 2020 for use in sedation during gastrointestinal endoscopy [7]. Currently, there is a wealth of research and clinical expertise available on the application of ciprofol in painless gastrointestinal endoscopy procedures. During a phase II clinical trial, all participants who received 0.2–0.5 mg/kg of ciprofol achieved successful sedation for colonoscopy [7,8]. Additionally, all doses were deemed safe and well tolerated, with no notable adverse events reported. During a study on gynecological surgery under general anesthesia, researchers observed that the ciprofol group had a lower overall incidence of adverse reactions than the propofol group (20 vs. 48.33%) [2].

For painless hysteroscopy, the use of strong anesthesia and sedation drugs is necessary. Since the method of anesthesia induction is comparable to that of painless gastrointestinal endoscopy, it is reasonable to consider that ciprofol could also be appropriate for hysteroscopy. On the other hand, hysteroscopy involves the use of dilators to expand the cervix and uterus intermittently throughout the procedure, leading to significant pain for patients. This pain can vary in intensity, requiring high doses of anesthesia to manage. However, these high doses may result in side effects like hypotension and respiratory depression when the pain subsides. Due to the specific nature of hysteroscopic surgery, it is essential to use anesthetic medications that have a mild impact on respiration and circulation. As a result, we have initiated a clinical trial to assess the efficacy of ciprofol in providing sedation for painless hysteroscopy, as well as to examine its effects on vital signs such as blood pressure, heart rate, and respiration in patients undergoing this procedure.

## 2. Materials and methods

### 2.1. Study design, ethics and trial registration

This is a prospective, single-center, randomized, non-inferiority study (Clinical Trial Registration Identifier: NCT06172140). The trial was conducted in accordance with the Declaration of Helsinki and Chinese clinical trial regulations. The study was approved by the Medical Ethics Committee of Sun Yat-sen Memorial Hospital, Sun Yat-sen University (approval number SYSKY-2023-1091-02), and all enrolled patients provided signed informed consent.

### 2.2. Participants

The participants of this prospective study were 124 women who planned to undergo hysteroscopy and treatment surgery under total intravenous anesthesia in the outpatient operating room from January 2024 to April 2024. The selection criteria were voluntary signing of an informed consent form, stable vital signs, ASA1-2 level, outpatients between the ages of 18 and 65 who had been evaluated by an anesthesia clinic. They were not allergic to anesthesia and had no contraindications to anesthesia. The exclusion criteria were inability or unwillingness to sign consent forms or inability to follow research procedures; patients with incomplete clinical data; anesthetic drug allergies; obvious central nervous system diseases, such as syphilis, brain tumors, cerebrovascular accidents, etc.; uncontrollable hypertension and diabetes; and a confirmed diagnosis of mental disorders by psychiatry, such as depression, physical and mental disorders, etc. They also had a history of using sedatives, hypnotics, anti-anxiety drugs, and antidepressants in the past 3 months.

### 2.3. Randomization

The patients were randomly assigned to the ciprofol group (Group C) or the propofol group (Group P) in a ratio of 1:1. The random number table, generated by an independent anesthesiologist using SPSS 26.0 software (IBM, Armonk, New York) was used to assign the random array. The randomized results were sealed in sequentially numbered envelopes until the end of the study. A nurse who was not involved in data collection or analysis was responsible for drug preparation. Both ciprofol and propofol are white emulsions and are digitally encoded. Therefore, the researchers responsible for postoperative follow-up and data processing did not know the grouping during the entire study period.

### 2.4. Procedures

Eligible patients undergoing hysteroscopy were randomly assigned in a ratio of 1:1 and were intravenously administered with ciprofol (0.4 mg/kg) (Haisco Pharmaceutical Group Co., Ltd., Liaoning, China) or propofol (2 mg/kg) (Kelun Pharmaceutical Group Co., Ltd., Sichuan, China). When the volumes of the two drugs were not equal, they were adjusted to the same volume using saline to ensure double blinding. All patients fasted for more than 8 h and were not allowed to drink water for at least 2 h before the operation. An 18-gauge cannula was placed in the dorsal vein of the hand to establish an intravenous injection channel. Pulse oxygen saturation (SpO_2_), blood pressure (BP) and heart rate (HR) were continuously monitored with a multi-parameter monitor (Mindray Biomedical Electronics Co., Shenzhen, China). Using the Capnostream 20p monitor (Medtronic, Minneapolis, MN, USA) to monitor the patients’ exhaled CO_2_ partial pressure and integrated respiratory index (IPI) and using a non-invasive dynamic respiratory monitoring device (Corespiron Medical Device Co., Ltd., Henan, China) (Supplementary Figure 1) to monitor the patients’ tidal volume (TV), respiratory rate (RR), and minute ventilation (MV). All patients received 5 μg of sufentanil (Yichang Humanwell Pharmaceutical Co., Ltd., Yichang, China) 1 min before intravenous infusion of ciprofol or propofol. The injection time of ciprofol or propofol was 30 s. When the Modified Observer’s Alertness/Sedation (MOAA/S) score was ≤1 [9], vaginal disinfection was started. During the induction of sedation, the MOAA/S score was evaluated every 30 s. If the MOAA/S score remained >1 for 2 min after the first use of the study drug, a supplementary dose of 1/2 of the initial dose was injected within 10 s. During the maintenance phase of anesthesia, the sedative was continuously infused intravenously at a rate of 1–1.5 mg/kg/h for ciprofol or 5–7 mg/kg/h for propofol. If there were signs of body movement, opening eyes, or speaking, a supplementary dose of half the initial dose was administered. If more than 5 supplementary doses were required within 15 min, painless hysteroscopy was considered to have failed. At this point, propofol (1 mg/kg) was used to strengthen sedation. In terms of sedative drug selection, propofol was the only alternative. During the operation, an oxygen flow rate of 5 L/min was continuously given through a nasal catheter. When the systolic blood pressure dropped below 90 mmHg or exceeded 30% of the baseline value, 1-2 mg of dopamine was injected intravenously. When the heart rate was less than 60 beats/min, 0.5 mg of atropine was injected intravenously. When SpO_2_ was less than 90%, the oxygen flow rate was increased to 10 L/min, and the lower jaw was lifted to open the airway. If the oxygen saturation could not rise back, face mask pressurized ventilation was actively carried out or a laryngeal mask was placed to control ventilation. The observation indexes were: (1) the success rate of hysteroscopy; (2) Induction time (MOAA/*S* ≤ 1 after the first dose); (3) Time for full recovery of consciousness; (4) Operation duration; (5) Additional times of anesthetics; (6) The incidence of hypotension, hypoxia, bradycardia, delayed awakening and injection pain; (7) Respiratory parameters and the minimum SpO2 and IPI values before anesthesia, after induction and after awakening.

### 2.5. Outcomes and data collection

The main result was the success rate of hysteroscopy: If more than five supplementary doses are required within 15 min, painless hysteroscopy is considered to have failed, and propofol is used to strengthen sedation.

#### 2.5.1. Secondary indicators and definitions

Induction time: the time from the beginning of propofol/ciprofol administration to MOAA/*S* ≤ 1 (seconds).

Examination duration: the time from the insertion of the vaginal speculum to the removal of the speculum at the end of the examination or operation (minutes).

Time for patient’s full recovery of consciousness: the time from the last propofol/ciprofol administration or speculum removal to five points for three consecutive MOAA/S scores (minutes).

Number of drug additions: the number of times the trial drug was added during the surgery.

Tidal volume: the volume of gas inhaled or exhaled each time during quiet breathing (mL).

Minute ventilation: The total amount of gas entering or leaving the lung per minute (L).

Respiratory rate: The number of breaths per minute (times).

Incidence of injection pain: When the patient reports pain or swelling at the injection site during administration, it is recorded as injection pain.

IPI value: The integrated pulmonary index. The respiratory detector is calculated based on the patient’s end-tidal carbon dioxide partial pressure (PETCO_2_), SpO_2_ and pulse rate. The reference values and significance are as follows.

**Table:** 

Index range	Patient status
10	Normal
8–9	Within normal range
7	Close to normal range; need attention
5–6	Need attention: intervention may be required
3–4	Need intervention
1–2	Need immediate intervention

#### 2.5.2. Safety index

Incidence of hypotension: When the systolic blood pressure is lower than 90 mmHg or drops more than 30% of the baseline value, it is recorded as hypotension.

Incidence of hypoxia: When the SpO_2_ is lower than 90%, it is recorded as hypoxia.

Incidence of bradycardia: Bradycardia is recorded when the heart rate is lower than 60 BPM.

Minimum SpO_2_: The lowest value of SpO_2_ from anesthesia induction to full consciousness of the patient.

Incidence of delayed awakening: 15 min after stopping administration, when the MOAA/S score is ≤3, it is recorded as delayed awakening.

### 2.6. Sample size and statistical analysis

The primary endpoint of the study was the success rate of painless hysteroscopy. The effect size for non-inferiority was reported as the absolute risk reduction (ARR), along with the 95% confidence interval (CI). An one-sided Type I error rate of 0.025 and a Type II error rate of 20% were chosen for the analysis. The sample size calculation was based on findings from a Phase IIb clinical study of ciprofol and other relevant studies, which suggested that the success rate for both groups was 98%. For non-inferiority, a margin of 8% was considered acceptable. To account for potential data loss of up to 20%, a total of 124 patients were required, with 62 patients in each group. SPSS 26.0 (IBM Corp., Armonk, NY, USA) was used for statistical analysis. Continuous variables were first evaluated for normality by the Shapiro–Wilk test and by visual inspection of Q–Q plots and histograms; homogeneity of variances was assessed with Levene’s test. Variables meeting both normality and equal-variance assumptions are reported as mean ± SD and compared between groups using Student’s *t*-test (employing Welch’s correction if variances proved unequal). Non-normally distributed variables are compared by the Wilcoxon rank-sum test. Categorical data are compared using the chi-square test or Fisher’s exact test, as appropriate. The primary endpoints were tested one-sided; all other comparisons were two-sided. A *p*-value ≤0.05 was considered statistically significant.

## 3. Results

### 3.1. General information

**Figure 1.**
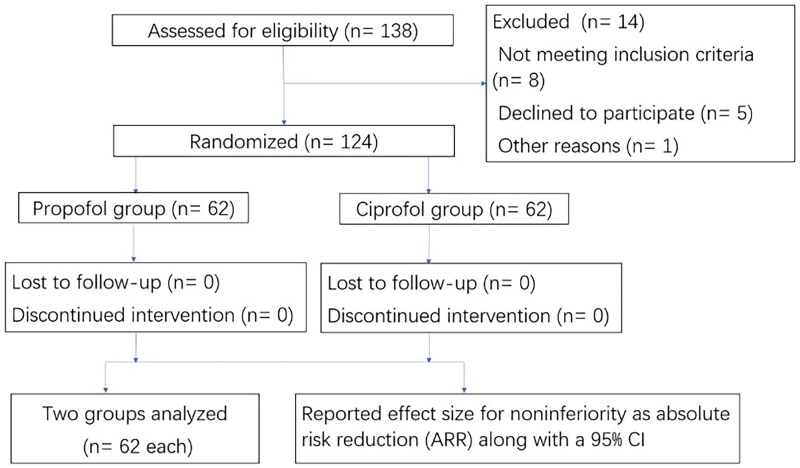
Flow chart of the study.

A total of 124 patients were included in the study, including 62 in the propofol group and 62 in the ciprofol group (Figure 1). There were no significant differences in age, height, or weight between the two groups (*p* > 0.05) (Table 1).

**Table 1.** Demographic and baseline characteristic of enrolled patients.

	Group (mean ± SD)		*p*
	Ciprofol (*n* = 62)	Propofol (*n* = 62)	
Age	35.313 ± 7.519	37.253 ± 7.508	0.102
Height	158.422 ± 4.961	157.266 ± 4.896	0.138
Weight	55.229 ± 7.580	54.614 ± 8.171	0.620
ASA class			0.828
Class I	48	49	
Class II	14	13	
Major comorbidities			0.797
Hypertension	8	6	
Diabetes	2	4	
Hyperthyroidism	2	2	
Hypothyroidism	2	1	
Reason for procedure			0.467
Endometrial polyps	28	24	
Intrauterine adhesion	34	38	

### 3.2. Preoperative physiological parameters

There was no significant difference in blood pressure, heart rate, SpO_2_, tidal volume, minute ventilation, respiratory rate, PETCO_2_ or IPI between the two groups before anesthesia (*p* > 0.05) (Table 2).

**Table 2.** Preoperative physiological parameters.

	Group (mean ± SD)		*t/z*	*p*
	Ciprofol (*n* = 62)	Propofol (*n* = 62)		
Systolic blood pressure (SBP)	126.084 ± 13.202	124.127 ± 13.743	0.925	0.356
Diastolic blood pressure (DBP)	76.398 ± 10.373	76.785 ± 10.721	0.234	0.816
Heart rate	75.892 ± 13.373	74.949 ± 11.599	0.478	0.633
SpO_2_	98.819 ± 0.814	98.734 ± 0.828	0.660	0.510
Tidal volume	409.627 ± 97.719	396.013 ± 83.985	0.949	0.344
Minute ventilation	6.500 ± 2.145	6.748 ± 2.276	0.714	0.476
Respiratory rate	17.049 ± 3.808	18.067 ± 3.629	1.740	0.084
PETCO_2_	34.518 ± 3.833	34.658 ± 3.096	0.255	0.799
IPI	9.795 ± 0.536	9.823 ± 0.525	0.331	0.741

### 3.3. Primary outcome

The success rate of hysteroscopy in both groups was 100%, with no difference between the two groups (Figure 1). All patients successfully underwent hysteroscopy without the use of alternative sedatives. The results showed that the success rate of anesthesia with 0.4 mg/kg of ciprofol was not lower than that with 2 mg/kg of propofol for painless hysteroscopy. The difference in success rate (ARR) between the two groups was 0% (95% CI −4.28 to 8.38%), indicating that the success rate of hysteroscopy under general anesthesia induced by ciprofol was not lower than that of propofol.

### 3.4. Intraoperative physiological parameters

The two groups were anesthetized according to the experimental scheme. When the depth of anesthesia reached the operation requirements, the blood pressure, heart rate, SpO_2_, tidal volume, minute ventilation, respiratory rate, PETCO_2_ and IPI values of each group were recorded again. The coefficient of variation of the data was used for weight assignment. Weights were assigned by measuring the relative dispersion of each indicator. A larger coefficient of variation indicates that the indicator carries more information, and should be assigned a higher weight. After calculating the mean and standard deviation for each indicator, the coefficient of variation is obtained, and weights are determined based on it, ultimately yielding the comprehensive index A (Table 3). An independent sample *t*-test was used to test the difference in the comprehensive index A between the two groups. It can be seen from the table that there was a significant difference in the comprehensive index A between the two groups (*t* = 3.100, *p* = 0.002) (Table 4).

**Table 3.** Calculation results of information weight method.

	Mean	Std. deviation	Coefficient of variation (%)	Weight (%)
Systolic blood pressure (SBP)	113.265	46.565	41.111	20.112
Diastolic blood pressure (DBP)	67.111	10.219	15.227	7.449
Heart rate	74.377	8.806	11.839	5.792
SpO_2_	97.833	1.991	2.036	0.996
Tidal volume	305.815	94.627	30.942	15.137
Minute ventilation	5.409	2.076	38.389	18.781
Respiratory rate	17.523	4.080	23.284	11.391
PETCO_2_	34.889	5.483	15.715	7.688
IPI	8.802	1.152	13.088	6.403
Minimum SpO_2_	91.975	11.753	12.778	6.251

**Table 4.** *T*-test analysis results of comprehensive index A, B, C.

	Group (mean ± SD)		*t*	*p*
	Ciprofol (*n* = 62)	Propofol (*n* = 62)		
Comprehensive index A	95.228 ± 18.485	87.299 ± 13.567	3.100	0.002**
Comprehensive index B	81.024 ± 10.033	75.969 ± 6.266	3.824	0.000**
Comprehensive index C	203.579 ± 50.173	186.267 ± 41.168	2.394	0.018*

Comprehensive indexes A, B, and C were based on the weighted methods mentioned above: A: Calculation results of the information weight method; B: Calculation results of the independence weight method; C: Calculation results of the CRITIC weight method. *p < 0.05; **p < 0.01.

Next, we will use the Independent Weighting Method to assign weights. The weights are allocated based on the correlation between indicators. If an indicator is highly correlated with other indicators, it suggests that the information it contains overlaps significantly with that of other indicators, and thus its weight should be lower. Conversely, if an indicator has weak correlations with others, it indicates that it contains more unique information, and its weight should be higher. After standardizing the data, the correlation coefficient matrix and multiple correlation coefficients are calculated to determine the weights and obtain the comprehensive index B (Table 5). The Independent Sample *t*-test was used to test the difference in comprehensive index B between the two groups. It can be seen from the table that there was a significant difference in comprehensive index B between the two groups (*t* = 3.824, *p* = 0.000) (Table 4).

**Table 5.** Calculation results of independence weight method.

	Multi-correlation coefficient (*R*)	Reciprocal of multi-correlation coefficient (1/*R*)	Weight (%)
Systolic blood pressure (SBP)	0.294	3.404	14.345
Diastolic blood pressure (DBP)	0.337	2.966	12.499
Heart rate	0.259	3.854	16.243
SpO_2_	0.412	2.427	10.230
Tidal volume	0.786	1.273	5.365
Minute ventilation	0.823	1.215	5.119
Respiratory rate	0.653	1.532	6.458
PETCO_2_	0.456	2.195	9.251
IPI	0.435	2.298	9.686
Minimum SpO_2_	0.390	2.564	10.805

Finally, we determined the weights of each indicator by taking into account both the contrast intensity and conflict of the indicators. The CRITIC weight method (Criteria Importance Through Intercriteria Correlation) is centered on utilizing the correlation between indicators and the variability of each indicator to reduce information overlap and more scientifically reflect the importance of each indicator. After standardizing the data, we calculated the standard deviation, correlation matrix, and conflict correlation indicators. The weights were then determined, and the comprehensive index C was calculated (Table 6). Independent sample *t*-test was used to test the difference in the comprehensive index C between the two groups. It can be seen from the table that there was a significant difference in the comprehensive index C between the two groups (*t* = 2.394, *p* = 0.018) (Table 4).

**Table 6.** Calculation results of CRITIC weight method.

	Variability	Conflict	Information	Weight (%)
Systolic blood pressure (SBP)	46.565	8.942	416.365	26.227
Diastolic blood pressure (DBP)	10.219	8.333	85.154	5.364
Heart rate	8.806	8.754	77.085	4.856
SpO_2_	1.991	8.809	17.543	1.105
Tidal volume	94.627	8.203	776.209	48.894
Minute ventilation	2.076	7.899	16.402	1.033
Respiratory rate	4.080	8.895	36.293	2.286
PETCO_2_	5.483	8.867	48.618	3.062
IPI	1.152	8.442	9.726	0.613
Minimum SpO_2_	11.753	8.860	104.129	6.559

After calculating and conducting two independent sample t-tests using three weight assignment methods, it was found that there was a significant difference in the comprehensive indicators between the two groups of patients. Therefore, we compared each indicator separately. It can be seen that the different groups of samples did not show significant differences in intraoperative systolic blood pressure, heart rate, tidal volume, respiratory rate, PETCO_2_, and IPI (*p* > 0.05). However, there was a significant difference in diastolic blood pressure, SpO_2_, minute ventilation, and minimum SpO_2_ between the two groups of patients after anesthesia (*p* < 0.05). A specific analysis shows that the ciprofol group had significantly higher diastolic blood pressure at the 0.01 level (*t* = 2.759, *p* = 0.006), with an average value of 69.23 mmHg compared to the propofol group, which had an average value of 64.89 mmHg. The ciprofol group also showed a significant difference at the 0.01 level (*t* = 3.711, *p* = 0.000) for SpO_2_, with an average value of 98.39%, compared to the propofol group’s average value of 97.25%. There was a significant difference in minute ventilation between the groups (*t* = 3.104, *p* = 0.002). The specific comparison showed that the average value of 5.89 L/min in the ciprofol group was significantly higher than the average value of 4.91 L/min in the propofol group. The difference in the minimum SpO_2_ during surgery between the two groups of patients was statistically significant (*t* = 2.906, *p* = 0.004), with an average of 94.58% in the ciprofol group, significantly higher than the average of 89.24% in the propofol group (Table 7). In summary, there were significant differences in four items: diastolic blood pressure, SpO_2_, minute ventilation, and minimum SpO_2_ during surgery.

**Table 7.** Intraoperative physiological parameters.

	Group (mean ± SD)		*t/z*	*p*
	Ciprofol (*n* = 62)	Propofol (*n* = 62)		
Systolic blood pressure (SBP)	120.012 ± 62.905	106.177 ± 14.709	1.906	0.058
Diastolic blood pressure (DBP)	69.229 ± 9.056	64.886 ± 10.935	2.759	0.006**
Heart rate	74.880 ± 8.742	73.848 ± 8.897	0.744	0.458
SpO_2_	98.386 ± 1.286	97.253 ± 2.404	3.711	0.000**
Tidal volume	318.747 ± 103.323	292.228 ± 83.032	1.795	0.075
Minute ventilation	5.887 ± 2.282	4.906 ± 1.710	3.104	0.002**
Respiratory rate	18.024 ± 4.234	16.996 ± 3.868	1.611	0.109
PETCO_2_	35.470 ± 5.467	34.278 ± 5.468	1.386	0.168
IPI	8.892 ± 1.036	8.709 ± 1.262	1.009	0.314
Minimum SpO_2_	94.578 ± 6.147	89.241 ± 15.185	2.906	0.004**

**p < 0.01.

**Figure 2.**
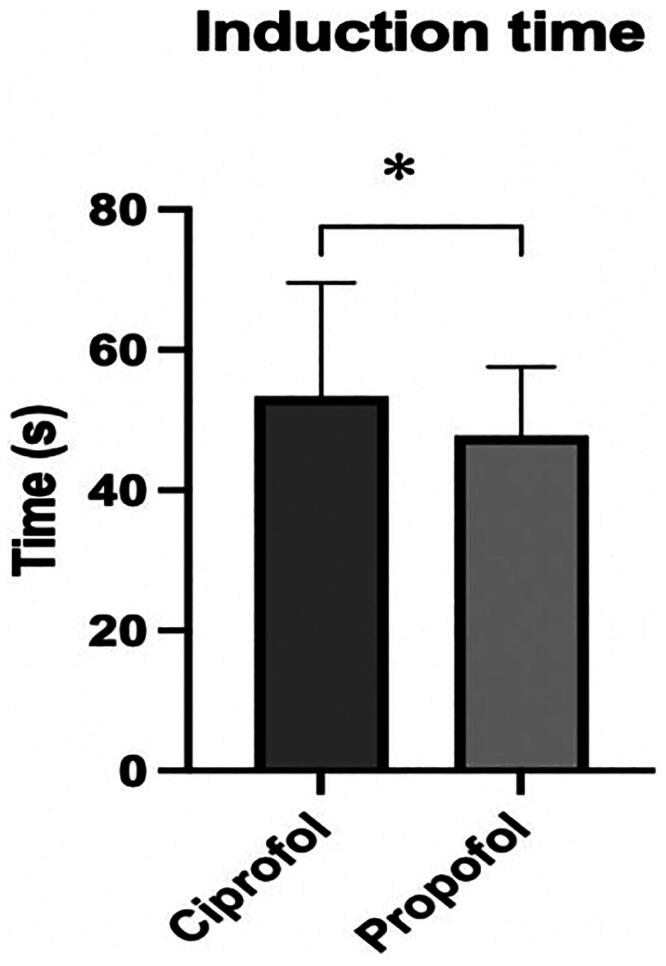
Induction time of the two groups. *p < 0.05; **p < 0.01

**Figure 3.**
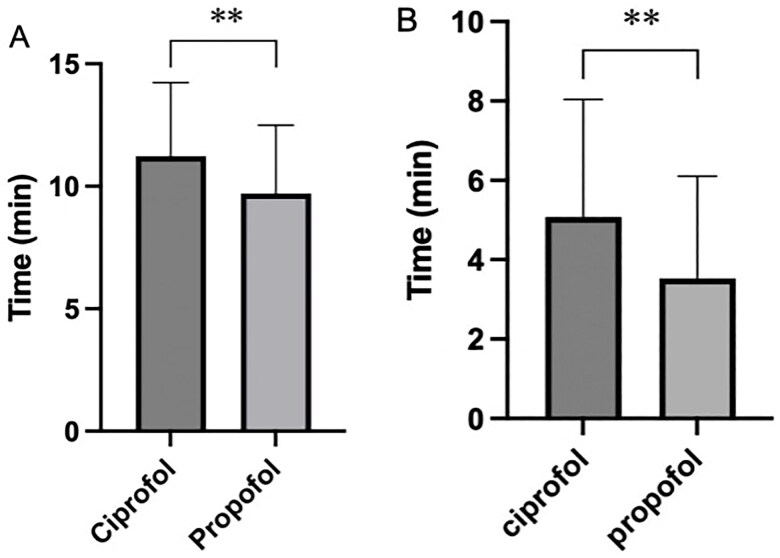
Time for full recovery of consciousness. (A) Time of last administration to recovery. (B) Time of hysteroscope withdrawal to recovery. The recovery time of patients in the propofol group was shorter than that of patients in the ciprofol group. *p < 0.05; **p < 0.01

There was no statistically significant difference in the duration of examination and the number of drug additions between the two groups of patients. However, there was a statistically significant difference in the induction time between the two drugs (*t* = 2.347, *p* = 0.021), with an average of 53.39 s in the ciprofol group and 47.74 s in the propofol group (Figure 2). We used two methods to calculate the time for patients’ full recovery of consciousness, and the results showed that the recovery time of patients in the propofol group was shorter than that of patients in the ciprofol group (Table 8, Figure 3).

**Table 8.** Induction, recovery, and drug addition.

	Group (mean ± SD)		*t*	*p*
	Ciprofol (*n* = 62)	Propofol (*n* = 62)		
Duration of examination (min)	18.482 ± 9.904	19.759 ± 8.881	0.863	0.389
Time of last administration to recovery	11.226 ± 3.010	9.710 ± 2.784	2.911	0.004**
Time of hysteroscope withdrawal to recovery	5.081 ± 2.955	3.532 ± 2.572	3.112	0.002**

**p < 0.01

### 3.5. Postoperative physiological parameters

After the operation, we continued to monitor the vital signs of the patients. The results showed that there was no significant difference in systolic blood pressure, diastolic blood pressure, heart rate, SpO_2_, tidal volume, minute ventilation, respiratory rate, PETCO_2_ and IPI between the two groups (*p* > 0.05) (Table 9).

**Table 9.** Postoperative physiological parameters.

	Group (mean ± SD)		*t/z*	*p*
	Ciprofol (*n* = 62)	Propofol (*n* = 62)		
Systolic blood pressure (SBP)	121.024 ± 16.751	131.139 ± 127.992	−0.714	0.476
Diastolic blood pressure (DBP)	76.289 ± 13.165	73.747 ± 12.111	1.277	0.203
Heart rate	71.325 ± 9.950	71.329 ± 9.207	−0.003	0.998
SpO_2_	98.289 ± 1.030	98.228 ± 1.085	0.369	0.713
Tidal volume	384.964 ± 92.385	390.886 ± 99.370	−0.393	0.695
Minute ventilation	6.358 ± 1.640	6.572 ± 1.768	−0.800	0.425
Respiratory rate	18.308 ± 7.381	18.470 ± 4.291	−0.169	0.866
PETCO_2_	36.506 ± 3.918	36.228 ± 4.652	0.412	0.681
IPI	9.506 ± 0.771	9.671 ± 0.796	−1.339	0.183

In addition, we also recorded the incidence of injection pain, perioperative hypotension, bradycardia, hypoxemia, and delayed recovery of patients in both groups. The results revealed significant differences in injection pain between the two groups. The proportion of patients who reported pain during intravenous anesthesia in the propofol group was 41.935%, which was significantly higher than the 1.613% in the ciprofol group (*p* < 0.05) (Table 10).

**Table 10.** Injection pain and safety index.

		Group (%)		Total (%)	*χ* ^2^	*p*
		Ciprofol (*n* = 62)	Propofol (*n* = 62)			
Injection pain	No	61 (98.387)	36 (58.065)	97 (78.226)	29.591	0.000**
	Yes	1 (1.613)	26 (41.935)	27 (21.774)		
Hypotension	No	58 (93.548)	55 (88.710)	113 (91.129)	0.898	0.528
	Yes	4 (6.452)	7 (11.290)	11 (9.259)		
Bradycardia	No	55 (88.710)	53 (85.484)	108 (87.097)	0.287	0.592
	Yes	7 (11.290)	9 (14.516)	16 (12.903)		
Hypoxia	No	44 (70.978)	37 (59.677)	81 (65.323)	1.744	0.187
	Yes	18 (29.032)	25 (40.322)	43 (34.677)		
Delayed awakening	No	59 (95.161)s	60 (96.774)	119 (95.968)	0.208	1.000
	Yes	3 (4.839)	2 (3.226)	5 (4.032)		

**p < 0.01.

## 4. Discussion

Propofol is commonly employed for induction and maintenance of general anesthesia. Its rapid onset and recovery also make it suitable for monitoring sedation in anesthesia care (MAC) [2,10]. However, propofol suppresses the circulatory and respiratory systems, potentially leading to induced hypotension, bradycardia, respiratory depression, and noticeable injection pain (with an incidence in adults ranging from 25 to 74%) [11–14]. More and more evidence show that intraoperative hypotension is related to a higher risk of organ damage, such as to the heart, kidneys and brain, as well as increased mortality in high-risk patients [15,16]. These adverse reactions restrict the use of propofol in clinical settings. Therefore, it is important for clinical anesthesiologists to consider alternative medications that can provide safety and comfort for patients during anesthesia induction without compromizsing the efficacy of anesthesia induction.

Ciprofol is a short-acting, 2,6-disubstituted phenol derivative propofol analog and a potentiator of the γ-aminobutyric acid type A receptor. Previous studies showed that there was no significant difference in anesthetic effect between 0.4 and 0.5 mg/kg of ciprofol and 2.0 mg/kg of propofol in colonoscopy [17], which was a Phase II multicenter clinical trial investigating ciprofol for anesthesia/sedation during colonoscopy. Our study protocol was based on its dosing regimen but focused on hysteroscopy and surgery due to the significant pain stimulus associated with hysteroscopy. While our findings were consistent with those of the referenced trial, our study expanded upon prior research by incorporating the monitoring of respiratory indicators such as tidal volume and PETCO_2_. This allowed for a more comprehensive safety assessment of ciprofol during intravenous general anesthesia with preserved spontaneous breathing. Currently, several other clinical trials have investigated the effectiveness and safety of ciprofol in patients undergoing examinations or surgeries under general anesthesia. During a study on anesthesia for gynecological daytime surgery, it was found that the ciprofol group experienced a longer time to lose consciousness than the propofol group. However, there was no statistically significant difference in awakening time between the two groups [18]. In a meta-analysis of 712 patients, it was found that the induction success rate, induction time and complete recovery time of ciprofol and propofol were comparable [19].

In our study, the success rate of hysteroscopy under general anesthesia induced by ciprofol was comparable to that of propofol. However, patients in the ciprofol group experienced a longer induction time (approximately 5.6 s) and postoperative recovery time (approximately 1.5 min) compared to those in the propofol group. Two factors may contribute to these differences. First, to maintain blinding during anesthesia induction, ciprofol was diluted with normal saline to match the volume of propofol. This dilution reduced the concentration of ciprofol, potentially leading to a slower onset of anesthesia. Second, the pharmacokinetic properties of ciprofol may also play a significant role. Ciprofol exhibits greater lipophilicity than propofol, making it more prone to accumulation in fatty tissues. This increased tissue retention affects both its distribution and elimination, which could explain the prolonged recovery time observed in the ciprofol group. Similar findings have been reported in previous studies, suggesting that lipophilic anesthetics may have delayed clearance due to redistribution effects [7,17,20]. Despite these differences, ciprofol demonstrated a non-inferior efficacy profile compared to propofol, with the lower limit of the 95% confidence interval (−4.28%) falling within the predefined non-inferiority margin. Furthermore, there was no significant variation in the occurrence of delayed awakening between the two groups in our study. We consider this slight prolongation in induction and recovery time to be tolerable in the majority of clinical settings.

The respiratory and circulatory inhibition of propofol has always been a major concern. Man [18] demonstrated that the propofol group experienced a more significant decrease in blood pressure and heart rate during gynecological surgery anesthesia compared to the ciprofol group. Additionally, a meta-analysis indicated that the ciprofol group had a lower occurrence of hypotension during anesthesia induction than the propofol [19]. However, the study of Peng [21] found that patients in both the ciprofol and propofol groups exhibited similar fluctuation trends in blood pressure, heart rate and SpO_2_ during anesthesia induction and intraoperative recovery, with no statistically significant differences. Our findings indicated that following anesthesia induction, patients in the ciprofol group had higher diastolic blood pressure, SpO_2_, minute ventilation and the minimum SpO_2_ value compared to those in the propofol group. Despite this, there was no significant variance in the occurrence of hypotension, bradycardia, and hypoxia between the two groups. Patients with no pre-existing medical conditions may experience mild respiratory and circulatory depression during anesthesia without serious consequences. However, for patients with underlying respiratory or cardio cerebrovascular conditions, fluctuations in vital signs due to anesthetics, could potentially lead to perioperative hypoxia or cardio cerebrovascular accidents. When hysteroscopic pain stimulation is reduced, the excessive amount of anesthetics administered to manage the previous pain may lead to a decrease in the patient’s oxygen saturation levels. Given the fact that we discovered that patients in the ciprofol group experienced higher minimum SpO_2_ values compared to those in the propofol group, it is possible that ciprofol could be a more ideal anesthetic for painless hysteroscopy in high-risk patients with underlying diseases. Nevertheless, further clinical research is necessary to confirm this potential advantage.

Another major challenge for anesthesiologists has been the pain associated with propofol injection. The incidence of pain during propofol injection was 45.1%, compared to only 8.8% for ciprofol [19]. The differing chemical compositions of propofol and ciprofol contribute to ciprofol’s lower lipophilicity. This tighter binding between ciprofol and the GABA receptor results in reduced plasma concentrations of ciprofol, ultimately explaining the decreased incidence of pain at the injection site [2,13,17,19,22,23]. Our research findings align with previous studies, showing that patients in the propofol group experienced a pain rate of 41.935% during anesthesia induction, which was significantly higher than the 1.613% reported by patients in the ciprofol group during intravenous injection of anesthetic drugs.

It is important to note that there is no significant difference in the Index of Patient Intolerance (IPI) between the two groups of patients following anesthesia. Our analysis showed that although there was a slight variation in oxygen saturation (SpO_2_) between the two groups after anesthesia, the levels were still within the normal range (with an average of 98.39% in the ciprofol group and 97.25% in the propofol group). Additionally, there was no significant difference in end-tidal carbon dioxide (PETCO_2_) levels and respiratory rate between the two groups. These factors contributed to the lack of difference in IPI values between the groups, with all values exceeding 8, indicating that the patients’ breathing did not require any additional support or intervention.

In summary, ciprofol is not inferior to propofol in terms of anesthetic efficacy during painless hysteroscopy. The upper limit of the 95% confidence interval (8.38%) slightly exceeds the predefined 8% non-inferiority margin, but the lower limit (−4.28%) remains within the margin, supporting the conclusion that ciprofol is non-inferior to propofol. In comparison to propofol, ciprofol has a relatively lower incidence of injection pain and causes less respiratory and circulatory depression in patients. However, its induction and recovery time are slightly longer than those of propofol. Nevertheless, the majority of the existing research has been carried out in China, and there is still a need for larger scale global studies to assess the efficacy and safety of ciprofol.

## 5. Conclusion

During painless hysteroscopy, ciprofol demonstrates non-inferiority to propofol in terms of anesthetic efficacy. Despite slightly longer induction and recovery times, ciprofol results in lower instances of injection pain and less disruption to patients’ respiration and circulation compared to propofol.

## 6. Limitations

This study demonstrates the non-inferiority of ciprofol to propofol—although the 95% CI upper limit (8.38%) slightly exceeds the predefined margin of 8%, the lower limit (−4.28%) remains within it. The exclusive focus on hysteroscopy limits applicability to other procedures. Moreover, because our primary aim was to evaluate ciprofol as a sedative alternative, we did not optimize the sufentanil regimen, which may have affected analgesic adequacy. We will conduct future studies to refine both sedative and opioid dosing and to evaluate pharmacokinetics, safety, and efficacy across varied patient populations and procedural settings.

## 7. Compliance with ethics guidelines

This article contains studies involving human participants performed by the authors and guidelines for ethical guidelines in research were followed. This article does not contain any studies involving animals performed by any of the authors.

## Supplementary Material

Supplemental Material

## Data Availability

Data sharing is available by emailing the corresponding author.
