# Supplementary figures and images for: Efficacy and safety of ciprofol for sedation/anesthesia in patients undergoing hysteroscopy: a prospective, randomized, non-inferiority trial
Source: Ann Med. 2025 Jun 18;57(1):2517820. doi: 10.1080/07853890.2025.2517820 (PMC12180349; doi:10.1080/07853890.2025.2517820)

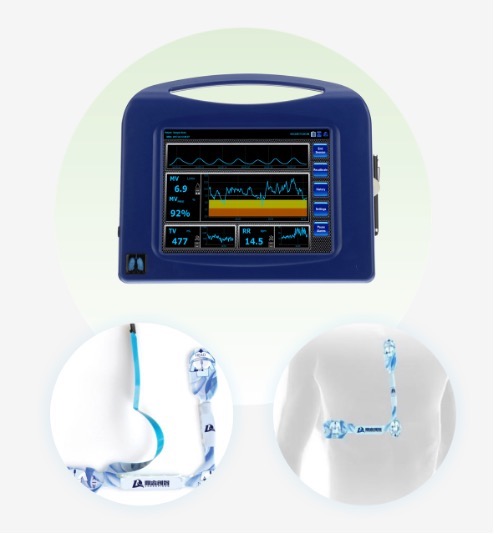

Supplement: Supplemental Material [file IANN_A_2517820_SM1746.jpg]
